# Association between perioperative prophylaxis with cefuroxime plus metronidazole or amoxicillin/clavulanic acid and surgical site infections in paediatric uncomplicated appendectomy: a Swiss retrospective cohort study

**DOI:** 10.1186/s13756-023-01312-1

**Published:** 2023-09-25

**Authors:** Isabella Bielicki, Hanna Schmid, Andrew Atkinson, Christian R. Kahlert, Christoph Berger, Nicolas Troillet, Jonas Marschall, Julia A. Bielicki, Swissnoso, Carlo Balmelli, Marie-Christine Eisenring, Stephan Harbarth, Didier Pittet, Hugo Sax, Matthias Schlegel, Alexander Schweiger, Laurence Senn, Rami Sommerstein, Sarah Tschudin Sutter, Danielle Vuichard Gysin, Andreas F. Widmer, Giorgio Zanetti, Walter Zingg

**Affiliations:** 1 Department of Paediatric Surgery, University of Basel Children’s Hospital, Basel, Switzerland; 2 Department of Infectious Diseases and Vaccinology, University of Basel Children’s Hospital, Spitalstrasse 33, 4056 Basel, Switzerland; 3 Paediatric Research Centre, University of Basel Children’s Hospital, Basel, Switzerland; 4 Department of Infectious Diseases, Bern University Hospital, Inselspital, University of Bern, Bern, Switzerland; 5 Infectious Diseases and Hospital Epidemiology, Cantonal Hospital St. Gallen, St. Gallen, Switzerland; 6 Infectious Diseases and Hospital Epidemiology, Children’s Hospital of Eastern Switzerland, St. Gallen, Switzerland; 7 Division of Infectious Diseases and Hospital Epidemiology, University Children’s Hospital Zurich, Zurich, Switzerland; 8 Service of Infectious Diseases, Central Institute, Valais Hospitals, Sion, Switzerland; 9 Swissnoso, The National Centre for Infection Control, Bern, Switzerland; 10 Division of Infectious Diseases, Department of Medicine, Washington University School of Medicine, St. Louis, MO USA; 11 Institute for Infection and Immunity, St George’s University of London, London, UK

**Keywords:** Perioperative antimicrobial prophylaxis, Surgical site infection, Paediatric surgery, Appendectomy, Comparative effectiveness analysis, Switzerland

## Abstract

**Objective:**

We aimed to evaluate the association between post-appendectomy SSI rates and the two most commonly used regimens for perioperative antimicrobial prophylaxis in Swiss children.

**Methods:**

We conducted a retrospective cohort study, analysing data from the Swiss national SSI surveillance database with a study period from 2014 to 2018. All hospitals undertaking paediatric appendectomies in Switzerland participate in the surveillance. We compared the cumulative incidence and odds of post-appendectomy SSI within 30 days of surgery in children ≤ 16 years of age undergoing appendectomy for uncomplicated appendicitis and receiving perioperative antimicrobial prophylaxis with cefuroxime plus metronidazole or with amoxicillin/clavulanic acid using multivariable adjusted logistic regression and propensity-score matching.

**Results:**

A total of 6207 cases were recorded in the study time frame. Overall SSI cumulative incidence was 1.9% (n = 119). 4256 children (54.9% male, median (IQR) age 12 [10, 14] years) received either cefuroxime plus metronidazole (n = 2348, 53.8% male) or amoxicillin/clavulanic acid (n = 1491, 57.0% male). SSI cumulative incidence was 1.1% (25/2348) among children receiving cefuroxime plus metronidazole and 2.8% (42/1491, *p* < 0.001) when receiving amoxicillin/clavulanic acid. The administration of cefuroxime plus metronidazole was associated with statistically significantly lower SSI odds compared to amoxicillin/clavulanic acid (aOR 0.35, 95%CI [0.20, 0.61], *p* < 0.001), and this was confirmed upon propensity-score matching.

**Conclusion:**

We found lower odds of post-appendectomy SSI in children receiving cefuroxime plus metronidazole compared to amoxicillin/clavulanic acid. Treating amoxicillin/clavulanic acid as the baseline, only 55 children need to receive cefuroxime plus metronidazole perioperative prophylaxis to avert one SSI. Existing guidelines recommending amoxicillin/clavulanic acid may need to be revised.

*Trial registration* ISRCTN47727811, registered retrospectively.

**Supplementary Information:**

The online version contains supplementary material available at 10.1186/s13756-023-01312-1.

## Introduction

Appendicitis is the most common reason for emergency abdominal surgery in children with an annual rate of 19–28 per 10,000 in children younger than 14 years [1–3]. In spite of current trends towards non-operative treatment of appendicitis[4], appendectomy remains the standard treatment in children with uncomplicated appendicitis [5]. Surgical site infection (SSI) remains one of the most common complications of appendectomy, occurring in about 6 out of 100 appendectomies in Europe [6].

SSIs are classified as nosocomial infections. The WHO estimates that up to 50% of such infections could be prevented [7]. One of the pillars of SSI prevention is the administration of single dose intravenous perioperative antimicrobial prophylaxis in patients undergoing surgery with expected bacterial contamination, including ‘clean contaminated’ and ‘contaminated’ operative wounds.[8]. In a large systematic review based on data from nearly 10,000 children and adults undergoing appendectomy, antimicrobial prophylaxis was concluded to be effective for SSI prevention [9].

Observational studies of adults undergoing colorectal surgery suggest the possibility of effectiveness of perioperative prophlyaxis for SSI prevention being related to the type of regimen being used [10, 11], but a recent systematic review and meta-analysis concluded that there was no difference in SSI between patients receiving cephalosporin and non-cephalosporin based regimens [12]. A range of regimens is recommended in continental Europe, with the use of a Penicillin antibiotic and a Beta-lactamase inhibitor preferred in France compared with a first or second generation cephalosporin plus metronidazole or an aminoglycoside in combination with clindamycin or metronidazole in Italy and the Netherlands [13–15].

The primary objective of this analysis was to compare the effectiveness of frequently used antibiotic regimes for SSI prevention in children and adolescents undergoing appendectomy for uncomplicated appendicitis in Switzerland.

## Methods

We hypothesised that a cephalosporin-based regimen may be more effective than a non-cephalosporin-based regimen, and that previous cohort studies addressing this question in paediatrics may have been underpowered to detect a difference in effectiveness[16–18]. Switzerland is a suitable setting to investigate this question, as variability in antibiotic use patterns by linguistic region mirroring that of Germany, France and Italy, respectively, is expected and a large relevant dataset is available for analysis [19].

### Study design and data collection

In this retrospective cohort study, we analysed data from the national SSI surveillance programme monitoring children who underwent appendectomy between 2014–2018. For appendectomies, all Swiss hospitals are required to collect standardized information on perioperative antimicrobial prophylaxis and outcome[20]. Demographic (gender, age) as well as procedure-related data (surgical approach, duration, American Society of Anesthesiologists (ASA) physical status classification, the degree of wound contamination, type of antibiotic(s) administered and timing of the administration of the first antibiotic) are collected at the time of intervention. The primary outcome SSI is assessed in a standardized manner 30 days after surgery by screening of the electronic health record and by structured telephone interview[21, 22]. We expect highly uniform SSI detection across Switzerland, as surveillance is organised at national level with regular training offered in all national languages. SSI is defined according to the Centers for Disease Control and Prevention NHSN criteria[23].

Data for patients 16 years of age and younger who underwent appendectomy between 01 January 2014 and 31 December 2018 in Switzerland were included. Patients older than 16 years as well as patients with complicated appendicitis (perforated appendicitis or established abscess, corresponding to an established infection requiring on-going post-operative antibiotic treatment) were excluded. Patients with missing procedure-related data were also excluded. Patients with incomplete data for receipt of antibiotics and timing of administration were included in the final analysis set, assuming that this represented lack of receipt of perioperative antimicrobial prophylaxis.

### Exposure of interest

The primary aim was to investigate the comparative effectiveness of the most frequently used cephalosporin-based regimen and non-cephalosporin-based regimen for preventing SSI. Patients not receiving any perioperative antibiotic prophylaxis were selected as an additional reference population. Of the available variables, sex, age, ASA classification, hospital where surgery took place, hospital size, type of surgical approach, duration of surgery and time of administration of perioperative prophylaxis before incision were all considered as potential confounders or covariates.

### Outcome of interest

The outcome of interest was SSI up to 30 days after appendectomy as captured within the Swiss national SSI surveillance programme.

### Statistical analysis

Descriptive data were analysed on all appendectomies performed, and the two most commonly used prophylactic antibiotic regimens were further evaluated.

Differences between the patients in the two antibiotic prophylaxis groups (cefuroxime plus metronidazole or amoxicillin/clavulanic acid) were investigated using descriptive statistics with the Kruskal–Wallis test for continuous variables, and the chi-square test (or variants thereof) for categorical variables.”Quantitative data are shown as medians (interquartile range [IQR]) and qualitative data as counts and percentages. Furthermore, we fitted uni- and multivariable logistic regression models with SSI as dependent variable, the antibiotic group, and risk factors timing of antibiotic prophylaxis, operation duration, sex, surgical approach (laparoscopic/open), ScoreT, hospital size (in beds) and ASA class as independent variables. Those variables significant at the 10% level in univariable models were then fitted in a multivariable model. The most parsimonious adjusted model was identified using forwards then backwards selection with the AIC as inclusion criterion. Sandwich-type standard errors were calculated to account for intra-hospital correlation. A supplementary analysis used 1:1 propensity score matching to identify pairs of similar patients from both groups, and then repeated the primary endpoint comparison (Additional file 1: eMethods 1).

A *p*-value of 5% was considered statistically significant unless stated otherwise. All analyses were performed using R version 3.6.1 [24].

### Ethics and study registration

All patients or their legal guardians are informed in writing regarding the data collection, with the option to actively withdraw their consent. The present study was approved by the local Ethics committee (Ethikkommission Zentral- und Nordwestschweiz EKNZ, Project ID 2018-02252). The study is registered with ISRCTN (ISRCTN47727811) The study is being reported in line with STROBE guidance (Additional file 2) [25].

## Results

**Fig. 1:**
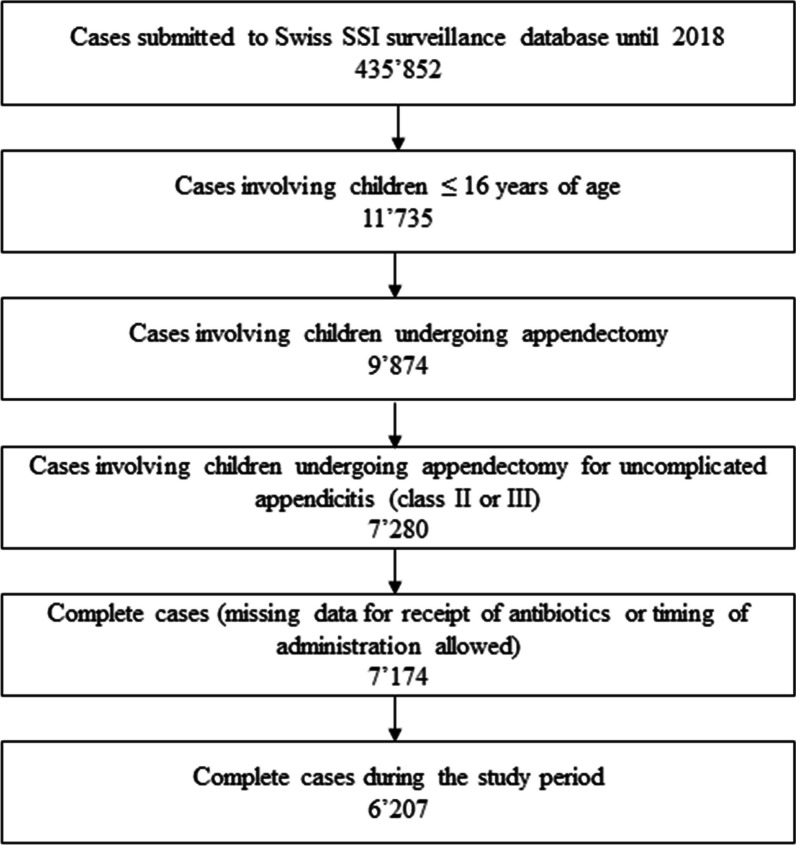
Flowchart of patient inclusion from the Swiss national SSI surveillance database

In the study time frame from 2014 to 2018, 7174 appendectomies were performed in children ≤ 16 years, of which 6207 had complete data (Fig. 1). Of these 119 (1.9%) experienced SSIs. Fifty-one different antibiotic regimens were administered as perioperative prophylaxis (Additional file 1: eResults 1). However, five regimens (cefuroxime plus metronidazole, amoxicillin/clavulanate, cefuroxime, cefazolin plus metronidazole and ceftriaxone plus metronidazole) and no antimicrobial prophylaxis accounted for the approach used in 90.7% of cases. Cefuroxime plus metronidazole and amoxicillin/clavulanic acid were the top two regimens jointly accounting for antimicrobial prophylaxis administered to 61.8% (3839/6207) of cases. No perioperative antimicrobial prophylaxis was used in 417 (6.7%) of cases.

Baseline demographic and clinical characteristics of the cases overall, and in the analysis cohort are shown in Table 1. Table 2 provides the same data in the analysis cohort by type of perioperative antimicrobial prophylaxis (cefuroxime plus metronidazole, amoxicillin/clavulanic acid, no antimicrobial prophylaxis). Of note, perioperative antimicrobial prophylaxis with amoxicillin/clavulanic acid was used more commonly in hospitals with higher bed numbers (200–499 beds), and children receiving amoxicillin/clavulanic acid had a shorter mean duration of surgery (92 min compared with 239 min for cefuroxime plus metronidazole).

**Table 1:** Baseline demographic and clinical characteristics for 6207 complete appendectomy cases recorded during the study period and for 4256 cases included in analysis

	Total surveillance cohort	Cases included in analysis
*Patient characteristics*		
Patients	6207	4256
Surgical site infections, n (%)	119 (1.9)	87 (2.0)
Sex, n (%)		
Male	3430 (55.3)	2336 (54.9)
Female	2777 (44.7)	1920 (45.1)
Age in years, median (IQR)	12 [10, 14]	12 [10, 14]
American Society of Anesthesiologists classification, n (%)		
Classes 1/2	6143 (99.0)	4220 (99.2)
Class 3/4/5	64 (1.0)	36 (0.8)
*Hospital characteristics*		
Hospital size, n (%)		
≤ 200 beds	2954 (47.6)	1943 (45.7)
200–499 beds	1563 (25.2)	1091 (25.6)
≥ 500 beds	1690 (27.2)	1222 (28.7)
*Procedure characteristics*		
Type of surgical approach n (%)		
Open	944 (9.4)	371 (8.7)
Laparoscopic	5623 (90.6)	3885 (91.3)
Duration of surgery in minutes, median (IQR)	53 [40, 68]	53 [40, 69]
Duration of surgery > 75th centile, n (%)	2151 (34.8)	1509 (35.5)
Perioperative antibiotic regimen, n (%)		
Cefuroxime plus metronidazole	2348 (37.8)	2348 (55.2)
Amoxicillin/clavulanic acid	1491 (24.0)	1491 (35.0)
Other regimen	1951 (31.4)	0 (0.0)
No antibiotics	417 (6.7)	417 (9.8)
Timing of administration of first prophylaxis prior to incision in minutes, median (IQR)	− 20 [− 38, − 10]	− 21 [− 40, − 11]

**Table 2:** Characteristics of 4256 appendectomy cases included in analysis by type of perioperative prophylaxis

	Cefuroxime plus metronidazole	Amoxicillin/clavulanic acid	No antimicrobial prophylaxis
*Patient characteristics*			
Patients, n	2348	1491	417
Infections n (%)	25 (1.1)	42 (2.8)	20 (4.8)
Sex, n (%)			
Male	1264 (53.8)	850 (57.0)	222 (53.2)
Female	1084 (46.2)	641 (43.0)	195 (46.8)
Age in years, median (IQR)	12 [10, 14]	12 [10, 14]	11 [10, 14]
ASA classification, n (%)			
Class 1/2	25 (1.1)	8 (0.5)	3 (0.7)
Class 3/4/5	2323 (98.9)	1483 (99.5)	414 (99.3)
*Hospital characteristics*			
Hospital size, n (%)			
≤ 200 beds	1158 (49.3)	560 (37.6)	225 (54.0)
200–499 beds	439 (18.7)	514 (34.5)	138 (33.1)
≥ 500 beds	751 (32.0)	417 (28.0)	54 (12.9)
*Procedure characteristics*			
Type of surgical approach n (%)			
Open	239 (10.2)	92 (6.2)	40 (9.6)
Laparoscopic	2109 (89.8)	1399 (93.8)	377 (90.4)
Duration of surgery in minutes, median (IQR)	56 [44, 72]	49 [39, 63]	53 [38, 65]
Duration of surgery > 75th centile, n (%)	966 (41.1)	400 (26.8)	143 (34.3)
Timing of administration of first prophylaxis prior to incision in minutes, median (IQR)	− 21 [− 35, − 12]	− 22 [− 73, − 10]	–

The comparison of the two antibiotic prophylactic regimes with those receiving no antibiotics confirmed a statistically significant higher SSI rate (4.8%, *p* < 0.001) in the absence of perioperative prophylaxis.

The univariable risk model identified a lower SSI risk when cefuroxime plus metronidazole rather than amoxicillin/clavulanic acid was administered (OR 0.37, 95% CI [0.21, 0.65], *p* =  < 0.001; Table 3). Older age was also marginally associated with lower risk (OR 0.94 [0.88, 1.0] for every year of age, *p* = 0.06).

**Table 3:** Crude and adjusted odds ratios (and 95% confidence intervals) from logistic regression evaluating associations between selected characteristics and post-appendectomy surgical site infection

	Crude OR (95% CI)	Adjusted OR (95% CI)
Sex		
Male	1.00	–
Female	0.71 (0.44, 1.16)	–
Age, for each additional year of age	0.95 (0.8, 1.01)	0.94 (0.88, 1.00)
ASA classification		
Class 1 or 2	1.00	1.00
Class 3, 4 or 5	3.71 (0.84, 16.41)	4.39 (0.99, 19.49)
Hospital size		
≤ 200 beds	1.00	–
200–499 beds	1.27 (0.65, 2.50)	–
≥ 500 beds	1.44 (0.74, 2.77)	–
Type of surgical approach		
Open	1.00	–
Laparoscopic	0.6 (0.24, 1.51)	–
Duration of surgery, for each additional 30 min	1.15 (1.00, 1.33)	1.22 (1.02, 1.46)
Duration of surgery > 75th centile	1.31 (0.80, 2.12)	–
Perioperative antibiotic regimen		
Amoxicillin/clavulanic acid	1.00	1.00
Cefuroxime plus metronidazole	0.37 (0.21, 0.65)	0.34 (0.19, 0.60)
Timing of administration of first prophylaxis prior to incision, for each additional 30 min	1.12 (1.03, 1.21)	1.11 (1.02, 1.20)

The following factors were found to be associated with a higher SSI risk (*p* < 0.1): timing of the first antibiotic administration more than 30 min before incision (OR 1.11 [1.02, 1.21] for every additional 30 min prior to incision, *p* = 0.01), surgery duration longer than 30 min (OR 1.15 [0.98, 1.34] for every additional 30 min, *p* = 0.09) and ASA levels 3/4/5 (OR 3.71 [0.84, 16.41] compared to levels 1/2, *p* = 0.09).

When fitted in the multivariable risk model, the administration of cefuroxime plus metronidazole was confirmed to be independently associated with lower SSI risk compared to amoxicillin /clavulanic acid (adjusted odds ratio (aOR) 0.34 [0.19, 0.60], *p* < 0.001; Table 3). Timing of antibiotic administration more than 30 min before incision and longer surgery duration were also identified as relevant risk factors for SSI, with older age and higher ASA score marginally significant at the 5% level (Table 3).

A supplementary propensity score matched analysis, which attempted to balance out potential differences between the patients in both groups confirmed the results (Additional file 1: eResults 2 and eResults 3).

## Discussion

Our multi-institutional analysis of 6207 children with uncomplicated appendicitis undergoing surgery supports the overall efficacy of preoperative antimicrobial prophylaxis to prevent SSI [26, 27]. Furthermore, the use of cefuroxime plus metronidazole was associated with a significantly lower rate of SSI compared to amoxicillin/clavulanic acid (1.1% vs. 2.8%). The considerable number and variability of different prophylactic regimens observed in this cohort alone likely reflect the lack of robust evidence on optimal regimens, which may instead be driven by surgeon preference and institutional consensus or guidelines.

To our knowledge, this is the first study in a paediatric population suggesting significantly lower odds of SSIs with a cefuroxime plus metronidazole-based prophylactic regimen. Considering other baseline characteristics in the cohort, we did not identify any obvious confounders for this observation. For example, open surgery was not more common in the amoxicillin/clavulanate acid group and duration of surgery was on average much shorter compared to the group receiving cefuroxime plus metronidazole.

Both combinations have been popular choices for antimicrobial prophylaxis in abdominal surgery and have been transferred from adult guidelines to paediatric surgery [8, 14, 15, 28, 29]. In the past amoxicillin/clavulanic acid may have been a preferred choice for antimicrobial prophylaxis in abdominal surgery, because of its superior coverage of enterococci, as well as for reasons of availability and cost-effectiveness compared to other antibiotic regimes [30, 31]. However, enterococcal infections are encountered less frequently after appendicitis than after biliary tract or colonic surgery [32]. Against this background, amoxicillin/clavulanic acid and cefuroxime plus metronidazole may be considered equivalent in their efficacy to prevent SSI in patients with uncomplicated appendicitis based on their expected antimicrobial spectrum.

Our study has several limitations. Since we cannot differentiate between those with missing values for their antibiotic prophylaxis (ie missing data) and those with confirmed “no prophylaxis”, the 3 group analysis including those without prophylaxis could potentially be biased. Furthermore, we lack data on the causative pathogens in cases of SSI in our study population. Microbiological evaluations are not usually undertaken at the time of appendectomy in cases of uncomplicated appendicitis. Similarly, empiric treatment of SSI without microbiological sampling and therefore data on causative organisms is the norm in the paediatric population [33]. Second, the observational nature of our study makes it susceptible to bias. We observe a greater proportion of amoxicillin/clavulanic acid use in midsize hospitals (200–499 beds). In Switzerland, these are likely to correspond to multi-disciplinary hospitals, in which paediatric surgical care may be offered by adult surgical services. In contrast, standalone paediatric hospitals, including tertiary institutions, all have ≤ 200 beds. Appendectomy being performed outside of a stand-alone paediatric hospital and potentially by general surgical teams without extensive paediatric expertise may therefore have biased findings towards higher SSI rates among children receiving amoxicillin/clavulanic acid. However, we observed shorter mean duration of surgery for children receiving amoxicillin/clavulanic acid. The longer mean duration of surgery in the cefuroxime plus metronidazole group suggests more complex cases in this group, for example young children and those primarily presenting with severe or atypical disease, being managed in paediatric surgical centres. Higher SSI rates would be expected among such patients. All in all, we expected clustering effects by hospital. We attempted to take this into account by propensity score matching patients. Replication of the observed associations in the propensity score matched analysis provides reassurance, but the case mix and operational procedures of different hospitals cannot be adjusted for definitively. Third, dosing of antibiotics used for perioperative prophylaxis may impact their efficacy, particularly if the dosing is not adjusted for patient weight. Information on administered doses is not collected as part of Swiss national surveillance; hence these data were not available for the current analysis. However, weight-based dosing is typical in paediatrics for all antibiotics administered intravenously, so that the impact of body weight may be expected to be limited. Moreover, information on administration of antibiotics more than three times or beyond 24 h after surgery is not collected as part of surveillance and was therefore not available. This could have further influenced the observed rate of SSI. We cannot rule out a differential effect between regimens, particularly with some indications that cefuroxime plus metronidazole may have been used more frequently in paediatric hospitals expected to manage more complicated cases. This may not be reflected adequately by ASA classifications in the cohort, as these are known to be less informative in children [34].

Nonetheless, given the size of the cohort and the high level of completeness and quality of data, our findings support recommending cefuroxime plus metronidazole over amoxicillin/clavulanic acid for antimicrobial prophylaxis in children undergoing appendectomy for uncomplicated appendicitis. This is in line with the recommendations in the first edition of the World Health Organization AWaRe Antibiotic Book [35].

Appreciating the relatively low incidence of SSIs with either of the two analysed prophylactic antibiotic regimens, the further risk reduction associated with cefuroxime plus metronidazole over amoxicillin/clavulanic acid (OR 0.35), may be considered a small effect on an individual patient level. However, starting with a baseline risk of 2.8% (amoxicillin/clavulanic acid), only 55 patients would need to be prescribed cefuroxime plus metronidazole to prevent a single SSI. The study therefore has important implications on efforts to improve quality of care and its findings could potentially be extrapolated to other types of childhood abdominal procedures.

Comparative effectiveness analyses based on real-world evidence, such as our study, may enable specific, clinically preferable regimens to be selected with greater confidence, especially if similar findings are replicated in different settings. As the incidence of SSI in children post appendectomy is low, conducting randomized controlled trials may be difficult and costly, and innovative and pragmatic platform designs may be more suitable. Future childhood SSI surveillance programs or other real-world datasets should consider including information on antibiotic dosing and body weight as well as timing of prophylaxis to guard against differences in effectiveness being due to inadequate dosing or incorrect timing of administration. Real-world evidence could also be complemented by translational research, for example investigating variations in the intestinal and oral microbiomes, including effects on the gut mucosal barrier, to understand the observed differences in SSI rates between different antimicrobial regimes with similar pathogen coverage.

## Conclusion

This is one of the largest paediatric cohorts analysing SSI rates after appendectomy for uncomplicated appendicitis over a period of 5 years. We find a significantly lower rate of SSI in children receiving cefuroxime plus metronidazole compared to amoxicillin/clavulanic acid. Despite the retrospective design, analysis of standardized surveillance data from many centres mitigating possible centre effects and confirmatory analysis using propensity score matching allows us to provide robust data. Based on our findings, guidance recommending the use of amoxicillin/clavulanic acid for surgical antimicrobial prophylaxis in children with acute uncomplicated appendicitis undergoing surgery may have to be revised in favour of cefuroxime plus metronidazole.

### Supplementary Information

**Additional file 1:** eAppendix.

**Additional file 2:** STROBE Statement.

## Data Availability

Anonymised data from mandatory Swiss national surveillance of surgical site infections can be obtained upon request by approaching the board of Swissnoso, the national centre for infection prevention, in writing.

## abbreviations

95% CI: 95% Confidence Interval
AIC: Akaike Information Criterion
aOR: Adjusted Odds Ratio
ASA: American Society of Anesthesiologists
IQR: Interquartile range
NHSN: National Healthcare Safety Network
OR: Odds Ratio
SSI: Surgical site infection
WHO: World Health Organization

## References

[1] Addiss DG, Shaffer N, Fowler BS, Tauxe RV (1990). The epidemiology of appendicitis and appendectomy in the United States. Am J Epidemiol.

[2] Anderson JE, Bickler SW, Chang DC, Talamini MA (2012). Examining a common disease with unknown etiology: trends in epidemiology and surgical management of appendicitis in California, 1995–2009. World J Surg.

[3] Ohmann C, Franke C, Kraemer M, Yang Q (2002). Status report on epidemiology of acute appendicitis. Chirurg.

[4] Minneci PC, Hade EM, Lawrence AE, Sebastião YV, Saito JM, Mak GZ (2020). Association of nonoperative management using antibiotic therapy vs laparoscopic appendectomy with treatment success and disability days in children with uncomplicated appendicitis. JAMA.

[5] Rentea RM, Peter SDS, Snyder CL (2017). Pediatric appendicitis: state of the art review. Pediatr Surg Int.

[6] Danwang C, Bigna JJ, Tochie JN, Mbonda A, Mbanga CM, Nzalie RNT (2020). Global incidence of surgical site infection after appendectomy: a systematic review and meta-analysis. BMJ Open..

[7] World Health Organization. Global Guidelines for the Prevention of Surgical Site Infection Geneva2018 [Available from: https://www.ncbi.nlm.nih.gov/books/NBK536404/.

[8] Bratzler DW, Dellinger EP, Olsen KM, Perl TM, Auwaerter PG, Bolon MK (2013). Clinical practice guidelines for antimicrobial prophylaxis in surgery. Surg Infect.

[9] Andersen BR, Kallehave FL, Andersen HK (2005). Antibiotics versus placebo for prevention of postoperative infection after appendicectomy. Cochrane Database Syst Rev..

[10] Deierhoi RJ, Dawes LG, Vick C, Itani KMF, Hawn MT (2013). Choice of intravenous antibiotic prophylaxis for colorectal surgery does matter. J Am Coll Surg.

[11] Ho VP, Barie PS, Stein SL, Trencheva K, Milsom JW, Lee SW (2011). Antibiotic regimen and the timing of prophylaxis are important for reducing surgical site infection after elective abdominal colorectal surgery. Surg Infect.

[12] Bowder AN, Yen CF, Bebell LM, Fernandes AR (2021). Intravenous cephalosporin versus non-cephalosporin-based prophylaxis to prevent surgical site infections in colorectal surgery patients: a systematic review and meta-analysis. Ann Med Surg.

[13] Bauer MP, van de Garde EMW, van Kasteren MEE, Prins JM, Vos MC. SWAB Richtlijn Peri-operatieve profylaxe: Stichting Werkgroep Antibioticabeleid; 2019 [Available from: https://swab.nl/en/preoperative-antibiotic-prophylaxis-general-information.

[14] Martin C, Auboyer C, Boisson M, Dupont H, Gauzit R, Kitzis M (2019). Antibioprophylaxie en chirurgie et médecine interventionnelle (patients adultes). Actualisation 2017. Anesth Réanim..

[15] per le Linee Guida - Programma Nazionale. Antibiotico profilassi perioperatoria nell'adulto LINEA GUIDA Data di pubblicazione: Settembre 2008 Data di aggiornamento: Settembre 2011. 2019.

[16] Bechis C, Michel F, Merrot T, Nicoleta P, Lando A, Leone M (2014). Comparison of two protocols of prophylactic antibiotic therapy in childhood appendectomy. Comparaison de deux protocoles d'antibioprophylaxie associee a l'appendicectomie de l'enfant.

[17] Cameron DB, Melvin P, Graham DA, Glass CC, Serres SK, Kronman MP (2018). Extended versus narrow-spectrum antibiotics in the management of uncomplicated appendicitis in children: a propensity-matched comparative effectiveness study. Ann Surg.

[18] Kashtan MA, Graham DA, Melvin P, Cameron DB, Anandalwar SP, Hills-Dunlap JL (2021). Ceftriaxone combined with metronidazole is superior to Cefoxitin alone in the management of uncomplicated appendicitis in children: results from a multicenter collaborative comparative effectiveness study. Ann Surg.

[19] Plüss-Suard C, Pannatier A, Kronenberg A, Mühlemann K, Zanetti G (2011). Hospital antibiotic consumption in Switzerland: comparison of a multicultural country with Europe. J Hosp Infect.

[20] Eisenring M, Perdrieu C, Berthod D, Troillet N. Teilnehmerhandbuch für das Modul Erfassung von postoperativen Wundinfektionen: Nationales Zentrum für Infektionsprävanton - Swissnoso; 2021 [updated 8th Dec 2021. Available from: https://www.swissnoso.ch/fileadmin/module/ssi_surveillance/Dokumente_D/1_Handbuch__Liste_der_Aenderungen__Definition/D_Version_01-10-2021_Teilnehmerhandbuch_Final.pdf.

[21] Kuster SP, Eisenring MC, Sax H, Troillet N (2017). Swissnoso. Structure, process, and outcome quality of surgical site infection surveillance in Switzerland. Infect Control Hosp Epidemiol..

[22] Troillet N, Aghayev E, Eisenring MC, Widmer AF (2017). Swissnoso. First results of the Swiss national surgical site infection surveillance program: who seeks shall find. Infect Control Hosp Epidemiol..

[23] Mangram AJ, Horan TC, Pearson ML, Silver LC, Jarvis WR (1999). Guideline for Prevention of Surgical Site Infection, 1999/ Centers for Disease Control and Prevention (CDC) Hospital Infection Control Practices Advisory Committee. Am J Infect Control..

[24] Team RC. R: A language and environment for statistical computing 2021 [Available from: https://www.r-project.org/.

[25] von Elm E, Altman DG, Egger M, Pocock SJ, Gotzsche PC, Vandenbroucke JP (2007). The strengthening the reporting of observational studies in epidemiology (STROBE) statement: guidelines for reporting observational studies. Lancet.

[26] Lee SL, Islam S, Cassidy LD, Abdullah F, Arca MJ (2010). Antibiotics and appendicitis in the pediatric population: an American Pediatric Surgical Association Outcomes and Clinical Trials Committee Systematic Review. J Pediatr Surg.

[27] Andersen BR, Kallehave FL, Andersen HK (2003). Antibiotics versus placebo for prevention of postoperative infection after appendicectomy. Cochrane Database Syst Rev..

[28] Senn Laurence, Vuichard Danielle, Widmer Andreas, Zanetti Giorgio, Kuster Stefan. Aktualisierte Empfehlungen zur perioperativen Antibiotikaprophylaxe in der Schweiz, 2015: Swissnoso; 2015 [Available from: https://www.swissnoso.ch/fileadmin/swissnoso/Dokumente/6_Publikationen/Bulletin_Artikel_D/v20_1_2015-09_Swissnoso_Bulletin_de.pdf.

[29] Paioni P, Aebi C, Bielicki J, Buettcher M, Crisinel PA, Kahlert CR (2022). Swiss recommendations on perioperative antimicrobial prophylaxis in children. Swiss Med Wkly.

[30] Dervisoglou A, Tsiodras S, Kanellakopoulou K, Pinis S, Galanakis N, Pierakakis S (2006). The value of chemoprophylaxis against enterococcus species in elective cholecystectomy: a randomized study of cefuroxime vs ampicillin-sulbactam. Arch Surg.

[31] Wilson AP, Shrimpton S, Jaderberg M (1992). A meta-analysis of the use of amoxycillin-clavulanic acid in surgical prophylaxis. J Hosp Infect..

[32] Sitges-Serra A, Lopez M, Girvent M, Almirall S, Sancho J (2002). Postoperative enterococcal infection after treatment of complicated intra-abdominal sepsis. J Br Surg.

[33] Zachariah P, Saiman L (2020). Expanding antimicrobial stewardship strategies for the NICU: Management of surgical site infections, perioperative prophylaxis, and culture negative sepsis. Semin Perinatol.

[34] Ferrari LR, Leahy I, Staffa SJ, Johnson C, Crofton C, Methot C (2020). One size does not fit all: a perspective on the American society of anesthesiologists physical status classification for pediatric patients. Anesth Analg.

[35] World Health Organization. The WHO AWaRe (Access, Watch, Reserve) antibiotic book. Geneva 2022. Available from: https://www.who.int/publications/i/item/9789240062382.

