# Supplementary material for: Association between perioperative prophylaxis with cefuroxime plus metronidazole or amoxicillin/clavulanic acid and surgical site infections in paediatric uncomplicated appendectomy: a Swiss retrospective cohort study
Source: Antimicrob Resist Infect Control. 2023 Sep 25;12:106. doi: 10.1186/s13756-023-01312-1 (PMC10521383; doi:10.1186/s13756-023-01312-1)
Supplement: Supplementary file 1 — Additional file 1: eAppendix. [file 13756_2023_1312_MOESM1_ESM.docx]

**eAppendix**

**Table of contents**

eMethods

eMethods 1: Propensity score analysis p 2

eResults

eResults 1: Overview of most common regimens used for perioperative prophylaxis p 2

eResults 2: Propensity score analysis – result of matching p 2

eResults 3: Results of crude and adjusted logistic regression in propensity-matched cohort p 3

**eMethods**

**eMethods 1: Propensity score analysis**

Rationale: We noted the imbalance between the hospital size following stratification by antibiotic prophylaxis group, and hypothesized that case mix (and therefore patient characteristics) might be different, potentially confounding the comparison made for the primary analysis.

Methods: We performed a post-hoc 1:1 propensity score matched analysis with 1491 patients on Cefuroxime plus metronidazole (group 1) and Amoxicillin/clavulanic acid (group 2). We included sex, age, operation duration, indicator variables for endoscopic (yes/no), overlong operation (yes/no), timing of the first antibiotic prophylaxis and hospital size to calculate the propensity score for being in groups 1 and 2. Matching was performed using a nearest neighbor algorithm. We then fit the primary analysis logistic regression model, once again adjusting for intra-hospital correlation using sandwich-type standard errors.

**eResults**

**eResults 1: Overview of most common regimens used for perioperative prophylaxis**

| **Antibiotic regime** | **n** | **%** | **Cumulative %** |
| --- | --- | --- | --- |
| Cefuroxime and metronidazole | 2348 | 37.8 | 37.8 |
| Amoxicillin/clavulanate | 1491 | 24.0 | 61.8 |
| Cefuroxime | 599 | 9.7 | 71.5 |
| Cefazolin and metronidazole | 427 | 6.9 | 78.4 |
| No antibiotic prophylaxis | 417 | 6.7 | 85.1 |
| Ceftriaxone and metronidazole | 350 | 5.6 | 90.7 |
| Cefazolin | 274 | 4.4 | 95.1 |
| Metronidazole | 135 | 2.2 | 97.3 |

Regimens accounting for >97% of drug utilization for perioperative prophylaxis among children undergoing appendectomy and used in at least 10% of cases are shown. Other regimens used in more than 10 children included cefuroxime and ornidazole, ceftriaxone, amoxicillin/clavulanate and metronidazole, cefamandole and metronidazole, cefepime and metronidazole, amoxicillin, clindamycin, cefazolin and ornidazole as well as ciprofloxacin and metronidazole. In total, 51 individual regimens were indicated as having been used for perioperative prophylaxis in uncomplicated appendectomy.

**eResults 2: Propensity score analysis – result of matching**

The matching process was successful (refer to Figure), with no large mean differences in the matched variables (not shown).

**Propensity score matched case and control patients**


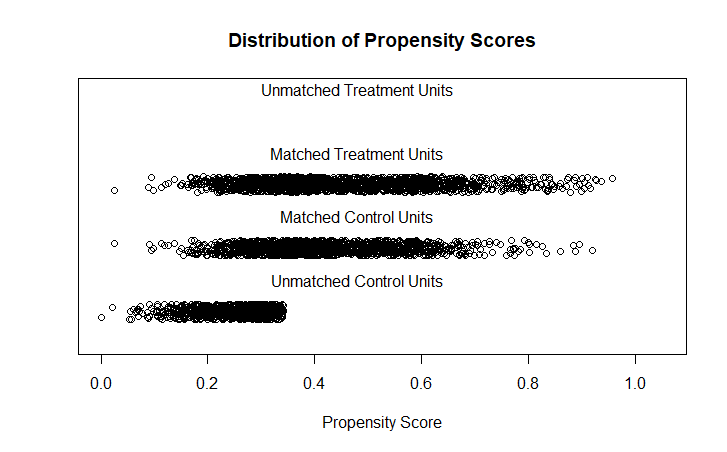


**eResults 3: Results of crude and adjusted logistic regression in propensity-matched cohort**

The difference between the antibiotic prophylaxis groups remained significant, confirming the primary analysis in the main manuscript (aOR 0.21, 95% CI [0.11, 0.41], p<0.001).

| **Variable** | | **crude OR (95%CI)** | **adjusted OR (95%CI)** |
| --- | --- | --- | --- |
| Sex | Male | 1.00 | - |
|  | Female | 0.78 (0.47, 1.28) | - |
| Age, for each additional year of age | | 0.95 (0.89, 1.02) | - |
| ASA classification | Class 1 or 2 | 1.00 | - |
|  | Class 3, 4 or 5 | 3.43 (0.46, 25.59) | - |
| Hospital size | ≤ 200 beds | 1.00 | - |
|  | 200-499 beds | 1.69 (0.71, 3.98) | - |
|  | ≥500 beds | 1.56 (0.53, 4.60) | - |
| Type of surgical approach | open | 1.00 | - |
|  | laparoscopic | 0.79 (0.25, 2.82) | - |
| Duration of surgery, for each additional 30 minutes | | 1.42 (1.05, 2.55) | 1.44 (1.07, 1.93) |
| Duration of surgery > 75th centile | No | 1.00 | - |
|  | Yes | 1.48 (0.78, 2.79) | - |
| Perioperative antibiotic regimen | Amoxicillin/clavulanic acid | 1.00 | 1.00 |
|  | Cefuroxime plus metronidazole | 0.21 (0.11, 0.40) | 0.21 (0.11, 0.41) |
| Timing of administration of first prophylaxis prior to incision, for each additional 30 minutes | | 1.13 (1.04, 1.24) | 1.12 (1.03, 1.21) |
